# Analysis of real-world scale-up processes for school-based mental health interventions

**DOI:** 10.1007/s10488-026-01491-0

**Published:** 2026-03-09

**Authors:** Kristel Jenniskens, Sanne Rasing, Daan Creemers, Arne Popma, Rixt Smit, Dominique van Pelt, Leonie van Vuuren, Saskia Mérelle, Jan Spijker, Femke van Nassau

**Affiliations:** 1 Behavioural Science Institute, Radboud University Nijmegen, Nijmegen, Netherlands; 2 GGZ Oost Brabant, Boekel, Netherlands; 3 Child and Adolescent Psychiatry & Psychosocial Care, Amsterdam University Medical Centers, Amsterdam, Netherlands; 4 Health Behaviors & Chronic Diseases, Amsterdam Public Health Research Institute, Amsterdam, Netherlands; 5 Department of Occupational Health, Amsterdam University Medical Centers, Amsterdam, Netherlands; 6 113 Suicide Prevention, Amsterdam, Netherlands; 7 Pro Persona, Nijmegen, Netherlands

**Keywords:** Implementation, Mental health, School, Prevention

## Abstract

The global rise in adolescent anxiety, depression, and suicidality has intensified calls for large-scale implementation of effective school-based interventions. This study examined determinants and strategies for scaling up school-based interventions targeting these mental health concerns. A mixed-methods approach was used, involving authors of published school-based mental health intervention trials. Of these, 45 authors completed a survey on scale-up decisions, intentions, and strategies. Determinants and strategies were further explored through 12 in-depth interviews. In our analysis of survey data, we identified interventions that were implemented on a small- or large-scale. In our analysis of interview data, we identified discontinued-, in the process of being scaled-up-, and scaled-up interventions. Half of the interventions surveyed reported intentions to scale up. Determinants for scale-up according to participants fell into three themes: (1) intervention characteristics (e.g., evidence base), (2) resources and support (e.g., availability of financial resources), and (3) school context (e.g., alignment with school curricula). The most relevant strategies for scaling up the included interventions were intervention registries for dissemination, adapting interventions to new settings, establishing organizational structures, and assessing/reducing costs. Our findings highlight strategies that potentially contribute to successfully scale-up of school-based mental health interventions. To enhance impact, we recommend applying such strategies – such as leveraging intervention registries and creating organizational support.

**Supplementary Information:**

The online version contains supplementary material available at 10.1007/s10488-026-01491-0.

## Plain Language Summary

More and more teenagers worldwide struggle with anxiety, depression, or suicidal thoughts. Schools can help by offering mental health programs, but these programs aren’t always available everywhere. This study looked at what it takes to successfully expand these programs to more schools. We asked researchers who had tested school mental health programs about their experiences. First, 45 researchers filled out a survey about their plans to expand (i.e., ‘scale’) their programs to other settings. Then, we interviewed 12 of them to learn what helps, or makes it harder, to scale these programs up. We found that for half of the programs, researchers planned to expand them and three things mattered most for success: 1) The program itself – it must work well and be easy to adapt; 2) Money and support – schools need funding and training; and, 3) School fit – the program should match the school’s needs and context. Helpful strategies for scaling up included: 1) adding the program to existing lists of proven interventions that schools and policy makers use when choosing interventions, 2) adapting the program for different school contexts, 3) having a team to help schools run the program; and, 4) keeping costs low. These findings are relevant to help reduce the number of teens with mental health problems. By looking at scaled-up programs, we were able to provide insight into what could help to expand effective programs across schools.

**Supplementary Information:**

The online version contains supplementary material available at 10.1007/s10488-026-01491-0.

## Introduction

Interventions promoting mental health and preventing mental illness help to improve population mental health (McGinty et al., 2024). The mental health of adolescents is currently a major concern: the prevalence of anxiety, depression and suicidality among adolescents is high (Biswas et al., 2020; Shorey et al., 2022; Uddin et al., 2019). Consequently, there has been a call to implement and scale-up effective mental health interventions (Dickson et al., 2022; Eaton et al., 2011; World Health Organization, 2019, n.d.). The World Health Organization (WHO) defines scale-up as ‘deliberate efforts to increase the impact of successfully tested health innovations so as to benefit more people and to foster policy and program development on a lasting basis’ (2010, p. 2). Multiple reviews and meta-analyses have shown that school-based interventions are effective in preventing anxiety, depression, and suicidal thoughts and behaviours in adolescents, and are appropriate for reaching a large population (Caldwell et al., 2021; Gijzen et al., 2022; Katz et al., 2013). Therefore, implementing successfully tested school-based mental health interventions on a larger scale seems promising.

Over the past decades, our understanding of how to describe, implement, and evaluate interventions in school settings has grown (Chambers & Emmons, 2024; Wilson et al., 2024). For instance, Cook et al. (2019) compiled a range of possible strategies for implementing health promotion interventions in school settings, and Baffsky et al. (2022) provided insight into the effectiveness of strategies for implementing school-based mental health interventions. Smith et al. (2022) found that effective replication of school-based health interventions into other settings requires adaptive strategies that provide facilitation to schools who are more reluctant to implement. Lawson and Azad (2024) recommend how to select and report implementation strategies for school-mental health, such as recognizing the importance of exploration and preparation for implementation success. Whilst our knowledge of implementation is growing, implementation often refers to activities within a single setting. Scale up, on the other hand, includes the broader, overarching activities and influences (e.g., political context) that affect expansion of programs to multiple settings on a larger scale. This distinction is made by others as well (McKay et al., 2019; Milat et al., 2015). Although we know that scale-up requires a different set of approaches and considerations than program implementation, our understanding of how to scale-up school-based mental health interventions remains limited (World Health Organization, 2010). With scale-up strategies, we refer to strategies aimed at increasing the number of settings that implement an intervention. The World Health Organization (2010) provides a framework for scale-up, in which scale-up strategies are distinguished regarding dissemination, organization of the scale-up process, cost and resource mobilization, and evaluation.

In public health, interventions seem to be rarely scaled-up (Dekkers & Luman, 2024; World Health Organization, 2010). Of those that are, the documentation of scale-up processes is often lacking (Eaton et al., 2011). In school-based mental health research specifically, whilst there are several scale up trials currently registered, the results are not yet available (Ochuku et al., 2023; Pathare et al., 2020; Werner-Seidler et al., 2020). To improve our understanding of scaling, this study aimed to evaluate attempts to scale up school-based mental health prevention interventions targeting anxiety, depression, and suicidality among youth. The following research questions were formulated:

- To what extent did school-based anxiety, depression and suicidality prevention interventions progress to scale-up after their initial trial period?
- What were the determinants for scaling up school-based anxiety, depression, and suicidality prevention interventions?
- Which strategies were used to scale-up school-based anxiety, depression, and suicidality prevention interventions?

## Methods

**Fig. 1:**
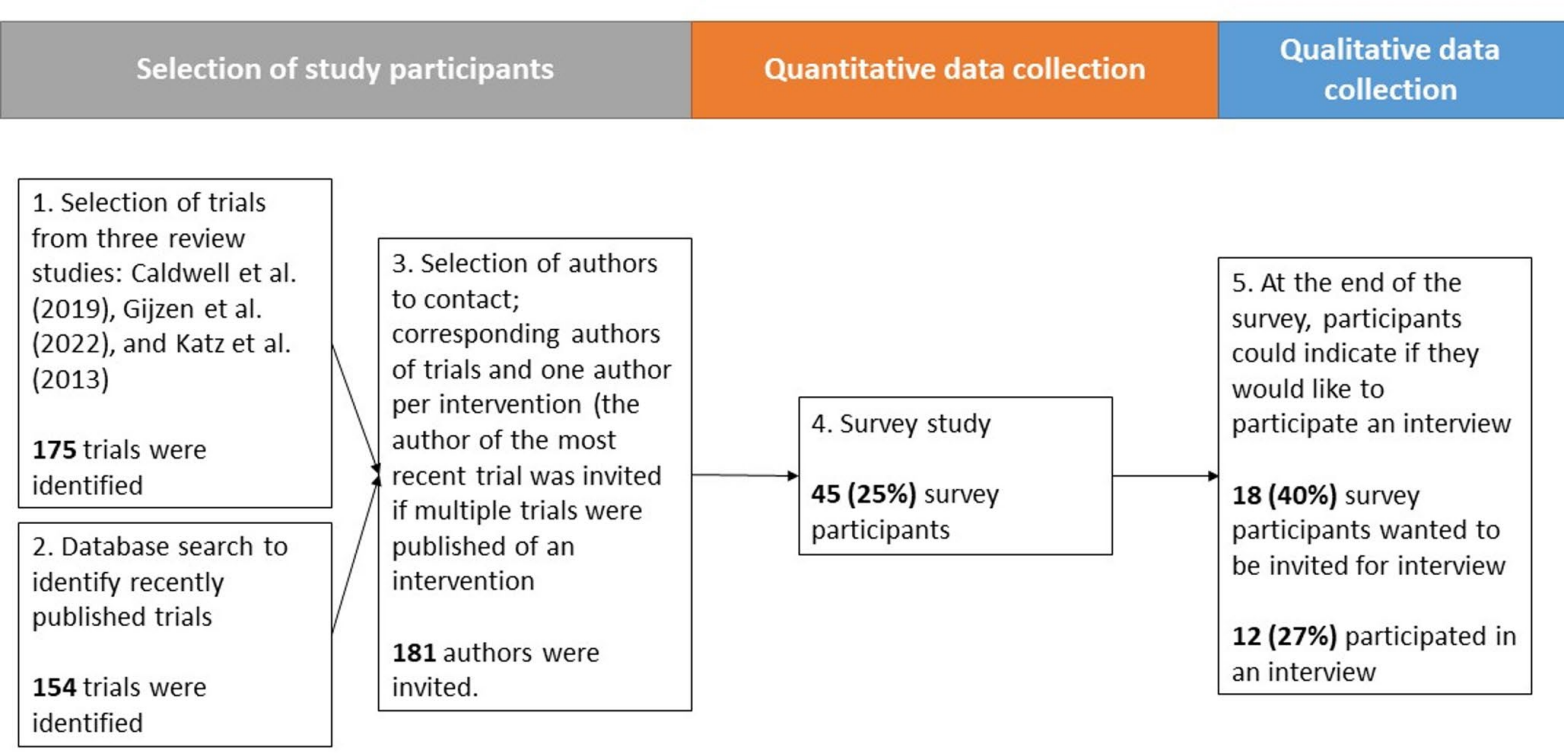
Sequence of sampling, and quantitative and qualitative research components

We conducted a sequential mixed-methods study of the processes involved in scaling up school-based interventions for preventing anxiety, depression, and suicidality. We started with a literature search to identify potential intervention trials, then contacted authors from identified papers with an invite to complete a survey, and then lastly collected qualitative data from a sub-sample of survey participants, to help explain our quantitative findings. Figure 1 provides an overview of the steps taken in conducting this study. Data were reported following the checklist for Mixed Methods Research Manuscript Preparation and Review (Lee et al., 2022) and the Standards for Reporting Qualitative Research (O’Brien et al., 2014).

### Study participants and recruitment

Participants included authors of school-based interventions trials for anxiety, depression, and suicide prevention. An overview of how we identified these authors is given in Fig. 1. We first identified trials (*n* = 175) that were included in review studies from Caldwell and colleagues (2019), Gijzen and colleagues (2022), and Katz and colleagues (2013). Next, we repeated the search strategies in Medline, PsycInfo, Embase, and Cochrane Central Register of Controlled Trials, to identify trials (*n* = 154) that were published after these three review studies were published. We applied the same search strategy to obtain an updated overview of intervention evaluation studies. We only included published trials to ensure that contacted authors would had sufficient involvement with and knowledge of the intervention from the start through to the end of the trial period, and could thus report on scale up. Studies were included if: (1) they reported a trial study, (2) the intervention was delivered in primary schools, secondary schools, vocational schools, and/or universities, (3) the intervention aimed to prevent anxiety, depression, and/or suicidality, (4) the target population was either children, adolescents, or students.

For interventions reported in multiple trial studies, we contacted the corresponding author of the most recent publication. As the aims of this study were to identify determinants of scale-up, authors were invited to complete the survey regardless of their intervention’s effectiveness. We identified 181 unique interventions for which we were able to invite the authors to participate.

Corresponding authors of included publications were invited to participate in the survey via email. Survey recruitment took place during April 2023 and a total of five reminders were sent every two weeks until June 2023. If the corresponding author was unable to be reached or did not respond, co-authors were contacted or other publicly available contact details were used (e.g. available through a google search). In total, 45 (25%) authors participated in the survey. On completion of the survey, participants were invited to participate in an online interview, and 12 (7%) participated.

### Data collection

#### Survey

The survey was administered via Qualtrics (Additional File 2). All survey participants provided active consent prior to participating. The first survey questions related to the intervention characteristics (i.e., first application, number of schools that implemented the intervention, intervention focus, and target population). The next survey questions related to participant characteristics, such as work experience and role in the intervention trial. The remaining questions related to whether the intervention was scaled-up after the trial.

Participants who indicated that their intervention was scaled up were asked about determinants influencing the decision to scale up and about scale-up strategies. These questions were based on the scale-up framework from Simmons and Shiffman (2007), which includes four domains of scale-up strategies.

1. Dissemination and advocacy through strategies such as cultivating champions of the intervention, publications or policy briefs.
2. Strategies for organizing scale-up and supporting implementation in potential settings, such as assessing needs in potential settings or building national capacity.
3. Strategies for cost and resource mobilization, such as cost assessments or applying for funding.
4. Strategies for monitoring and evaluation of the scale-up process, such as tracking systems and adjusting scale-up strategies according to evaluation outcomes.

Survey participants were asked to select up to three strategies per domain. Finally, we asked whether the intervention was scaled locally, regionally, nationally, or internationally.

Participants who indicated that their intervention was not scaled up were asked about determinants influencing this decision, based on the survey questions from (Smit et al. 2024a, b). Additionally, we asked whether there was interest in scaling up and, if so, which stakeholders were interested.

#### Interviews

Survey respondents who indicated that they would be willing to participate in an online interview, were contacted via email and provided signed informed consent prior to the interview commencing. Online semi-structured interviews were conducted by the lead author (KJ) via Microsoft Teams, between May and August 2023. KJ has experience in conducting qualitative interviews. There were no prior relations between the interviewer and interview participants. Interviews followed a topic list (Additional file 3), which included ‘participant & intervention’ and ‘scale-up decisions’, and ‘scale-up processes’ and ‘scale-up success’. After each interview, participants received a summary for member-checking.

### Data analysis

#### Survey

Responses with missing data were excluded if the participant only completed the section relating to the ‘intervention’ and ‘participant characteristics’. Survey data were analysed by calculating descriptive statistics in SPSS. To differentiate between the level of scale-up, we categorised interventions as scaled up on either a large scale (implemented in >10 schools) or small-scale (implemented in 1-10 schools). As there is no clear cut-off point for when an intervention can be considered to be implemented at scale, we chose 10 schools as a cut-off point as this led to a roughly equal distributions of interventions across the categories small and large scale.

#### Interviews

Interview transcripts were analysed thematically in Atlas.ti. Thematic analysis was used to identify recurring themes related to scale-up in school settings. First, eight transcripts were open-coded by three researchers (KJ, RS, DP) to ensure triangulation. These researchers then categorized their codes through axial coding, and a code tree was developed in agreement between all three researchers (Additional File 4). Main themes identified were based on the scale-up framework from Simmons and Shiffman (2007): “scale-up barriers,” “scale-up facilitators,” “strategies for dissemination and advocacy,” “strategies for organizational processes,” “strategies for mobilizing costs and resources,” and “strategies for evaluation and monitoring.” Next, the lead author (KJ) coded all transcripts deductively using the code tree. Three researchers (KJ, FN, SR) reviewed the analyses and regrouped codes into three themes, with corresponding subthemes: 1) decision to scale-up, 2) scale-up determinants (i.e. intervention characteristics, resource availability, and school context), and 3) scale-up strategies (i.e. dissemination and advocacy, organizational process, mobilizing costs and resources, and evaluation).

## Results

From the 181 authors contacted, 45 authors (25%) completed the survey, and 12 (27%) of survey respondents participated in an interview (Fig. 1). Most participants worked in North America (33% for both survey and interviews) or Europe (22% survey, 33% interviews), and worked in academia during the intervention trial (82% survey, 58% interviews). An overview of participant characteristics is presented in Additional File 5. Secondary school students were the most common target population in intervention trials (73% survey, 58% interviews). From survey data, 47% of interventions were ongoing projects and 56% were in the process of being scaled-up. Most interventions were reported as effective in reducing anxiety, depression, or suicidality, or on related variables, such as help-seeking behaviour or mental health literacy (89% of surveys, 83% of interviews). An overview of the interventions included in this study is presented in Table 1.

**Table 1:** Intervention characteristics

		**Interventions from included publications ** **(n = 181)**	**Interventions reported on in the survey** **(n = 45)**			Interventions reported on in the interviews ** **(n = 15)**			
			Total (n = 45)	Small-scale interventions (1-10 schools) (n = 18)	Large-scale intervention (>10 schools) (n = 13)	Total (n = 12)	Discontinued interventions (n = 6)	In-progress examples (n = 3)	Success examples (n = 3)
**First applied in practice**	Before 2004		10 (22%)			3 (25%)			
	2004-2008		13 (29%)			1 (8%)			
	2009-2013		8 (18%)			0 (0%)			
	2014-2018		10 (22%)			4 (33%)			
	2019-2024		9 (20%)			4 (33%)			
	Unknown by authors		3 (7%)			0 (0%)			
**Focus of the intervention**	Anxiety	73 (40%)	15 (33%)			3 (25%)			
	Depression	95 (52%)	17 (38%)			5 (42%)			
	General mental health	11 (6%)	11 (24%)			2 (17%)			
	Suicidality	43 (24%)	12 (27%)			4 (33%)			
	Other	6 (3% )	12 (27%)			1 (8%)			
**Target population**	Primary school students	36 (20%)	10 (22%)			4 (33%)			
	Secondary school students	93 (51%)	33 (73%)			7 (58%)			
	Vocational school students	1 (1%)	4 (9%)			0 (0%)			
	University students	27 (15%)	12 (27%)			0 (0%)			
	Other	24 (13%)	7 (16%)			1 (8%)			
	Unknown	19 (10%)	0 (0% )			0 (0%)			
**Ongoing project**	Yes		21 (47%)			6 (50%)			
	No		24 (53%)			6 (50%)			
**Intention/efforts to scale-up**	Yes		25 (56%)	7 (39%)	10 (77%)	7 (58%)	1 (17%)	3 (100%)	3 (100%)
	No		20 (44%)	11 (61%)	3 (23%)	5 (42%)	5 (83%)	0 (0%)	0 (0%)
Intervention effects found in the trial*	Yes		40 (89%)	16 (89%)	12 (92%)	10 (83%)	4 (67%)	3 (100%)	3 (100%)
	No		5 (11%)	2 (11%)	1 (8%)	2 (17%)	2 (33%)	0 (0%)	0 (0%)

* Based on the methods and result sections in the trial publication

** Interviewees were categorized as success if efforts to scale-up were ongoing, and >10 new settings had already implemented their intervention after the trial. Interviewees were categorized as an in-progress if it was an ongoing project and there was an intention to scale-up, but ≤10 settings implemented their intervention after the trial. Interviewees were categorized as discontinued if their intervention was no longer implemented

### Extent to which interventions were scaled-up

Of the scaled up interventions reported on in the survey (*n* = 25), 7 were classified as small-scale (28%), 10 were classified as large-scale (40%), and for 8 interventions the scale was unknown (32%). Of the interventions discussed in the interviews, most were discontinued (50%), three (25%) were classified as ‘in-progress examples’, and three (25%) were classified as ‘scaled-examples’. An overview of all intervention characteristics is provided in Table 1.

### Decision to scale up

**Fig. 2:**
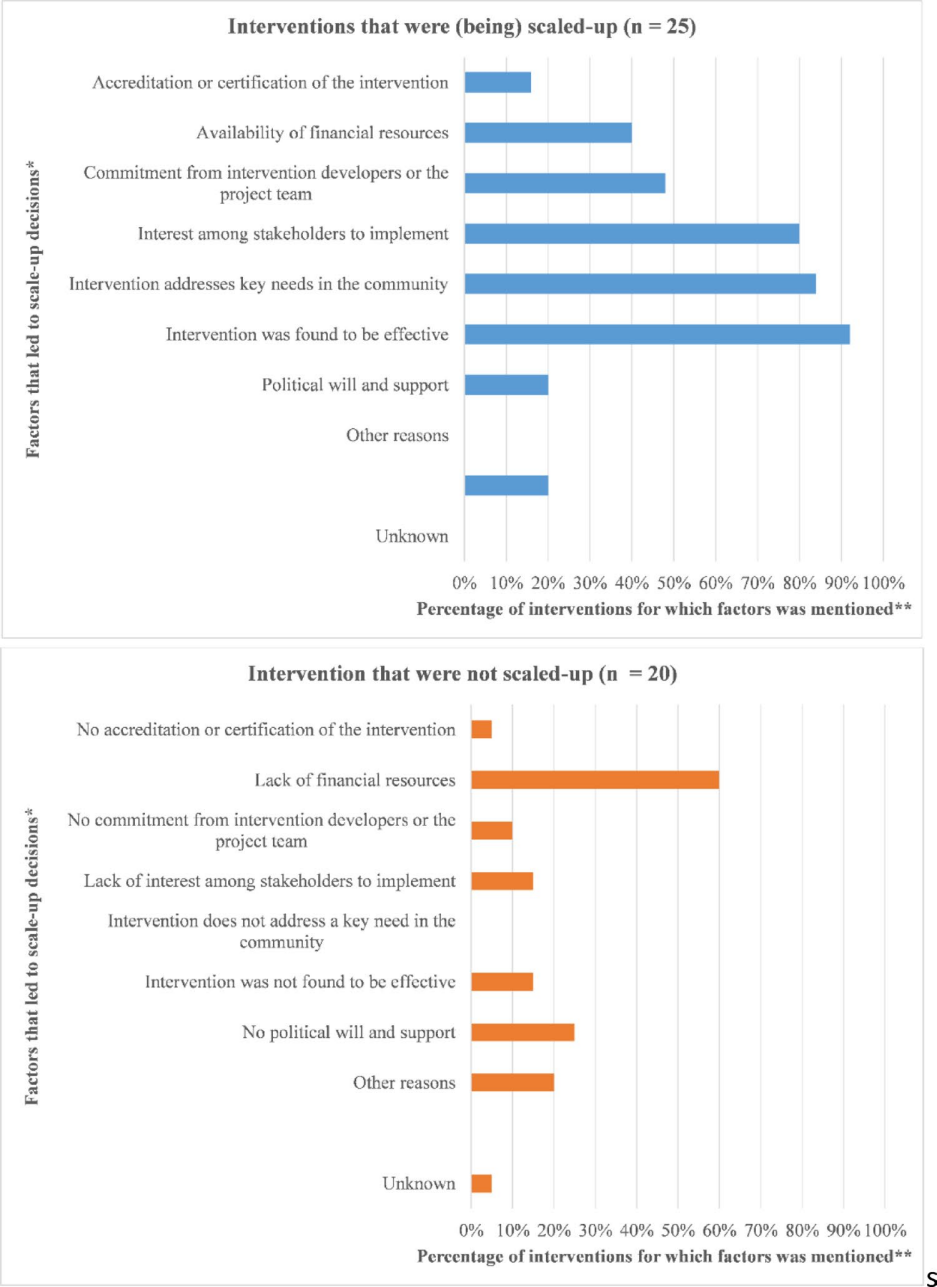
Determinants leading to scale-up decisions based on survey data. * As reported by survey participants.** Survey participants could select multiple answers

For most interventions reported on in the survey, there was an interest or intention to scale-up (56%), mainly from intervention developers (50%) and the trial research team (45%). Additionally, we asked about determinants that influenced the scale-up decision (Fig. 2). The most prevalent factor that led to a decision to scale-up was if the intervention was found effective (92%), followed by the intervention addressing a key need in society (84%) and an interest among stakeholders to scale-up (80%). The most frequently mentioned factor leading to a decision not to scale-up was a lack of financial resources (60%), followed by no political will or support for scale-up (25%).

The interviewees mentioned that for most interventions, there was no clear ‘decision moment’ for scale-up. Participants explained it as an iterative process of expanding the scale at which the intervention was offered.

### Determinants to scale up

Interview participants provided insights into determinants that hindered or facilitated scale up of their interventions across three themes: intervention characteristics, resource availability, and school context. An overview of the determinants per theme is provided in Table 2. The most relevant interview quotes supporting these results are provided in text. Additional quotes are presented in Additional File 6.

**Table 2:** Scale-up barriers and facilitators based on interview data

	Barriers	Facilitators
Intervention characteristics	- Lack of evidence for intervention effectiveness - No fit with political agendas	- Evidence for intervention effectiveness - Intervention less time-intensive than alternatives - Fit with societal needs
Resource availability	- Lack of financial resources for scale-up - Staff shortages in education and healthcare	- Availability of financial resources for scale-up - Committed project team
School context	- Intervention does not fit into school curricula - Lack of knowledge about effective mental health prevention among stakeholders in education	

#### Intervention characteristics

Based on both survey and interview data, the most frequently reported factor influencing decisions to scale-up was effectiveness of the intervention (92%, Fig. 2). Moreover, some interventions were discontinued due to a lack of evidence for their effectiveness.

*“In 2010 the first RCT was published […] and that was a catalyst that put [the intervention] on the map.”* – Scaled-example 1, North America.

Other intervention characteristics that facilitated scale-up of the included interventions were that interventions were less intensive compared to other programs, and that the interventions addressed an urgency felt in society to prevent mental health problems and suicidality in young people.

*“Some schools were really interested in mental health*,* because*,* unfortunately*,* there were a lot of suicides in a school*,* and they wanted to do something about that*.” – Scaled-example 2, North America.

In addition, a lack of political support for scaling up mental health interventions was a barrier for multiple discontinued interventions and for those interventions that were in progress of being scaled up.

*“If the Ministry [of Education] doesn’t sponsor or develop an intervention itself*,* it doesn’t generally support external interventions coming in.”* – discontinued intervention 2, Oceania.

#### Resource availability

Regarding the second theme ‘Resource availability’ (Table 2), the survey data showed that for the included interventions, the most important factor that influenced decisions to not scale-up was lack of financial resources (60%). This is supported by the interview data: for most discontinued interventions and in-progress examples, a lack of financial resources hindered scale-up. Likewise, the availability of funding for scale-up was a facilitator for the scaled-examples.

*“You get money for a period [to trial the intervention], but then it’s over and you don’t get anything to scale-up.”* – discontinued intervention 3, Europe.

Another resource related barrier for most interventions, including scaled-examples, were staff shortages in education and healthcare. For some, this was an important reason why the intervention had to be discontinued.

*“Currently a barrier to scalability is underfunded public school systems*,* decrease in educators*,* and lack of substitutes.”* – Scaled-example 1, North America.

Additionally, individuals involved in the scale up process were related to scale-up success for the example cases in our study. For two of the scaled-examples, having committed individuals in the project team facilitated scale-up.

*“Our founder had a vision for scaling it nationally.”* – Scaled-example 1, North America.

#### School context

Most interview participants reported barriers for scale-up that related to the school context (Table 2). This included that most schools did not prioritize mental health prevention.

*“[Schools] have priorities in academics and learning*,* so where to fit [our intervention] is often a challenge for them.”* – Scaled-example 1, North America.

Some interview participants found that a lack of knowledge about effective mental health prevention among stakeholders in education, such as policy makers and school boards, hampered scale-up.

*“There is a misunderstanding in our Ministry of Education about the difference between mental health literacy and mental health promotion. […] They think just learning the words and language is enough*,* but it is not.”* – discontinued intervention 2, Oceania.

### Scale-up strategies

**Fig. 3:**
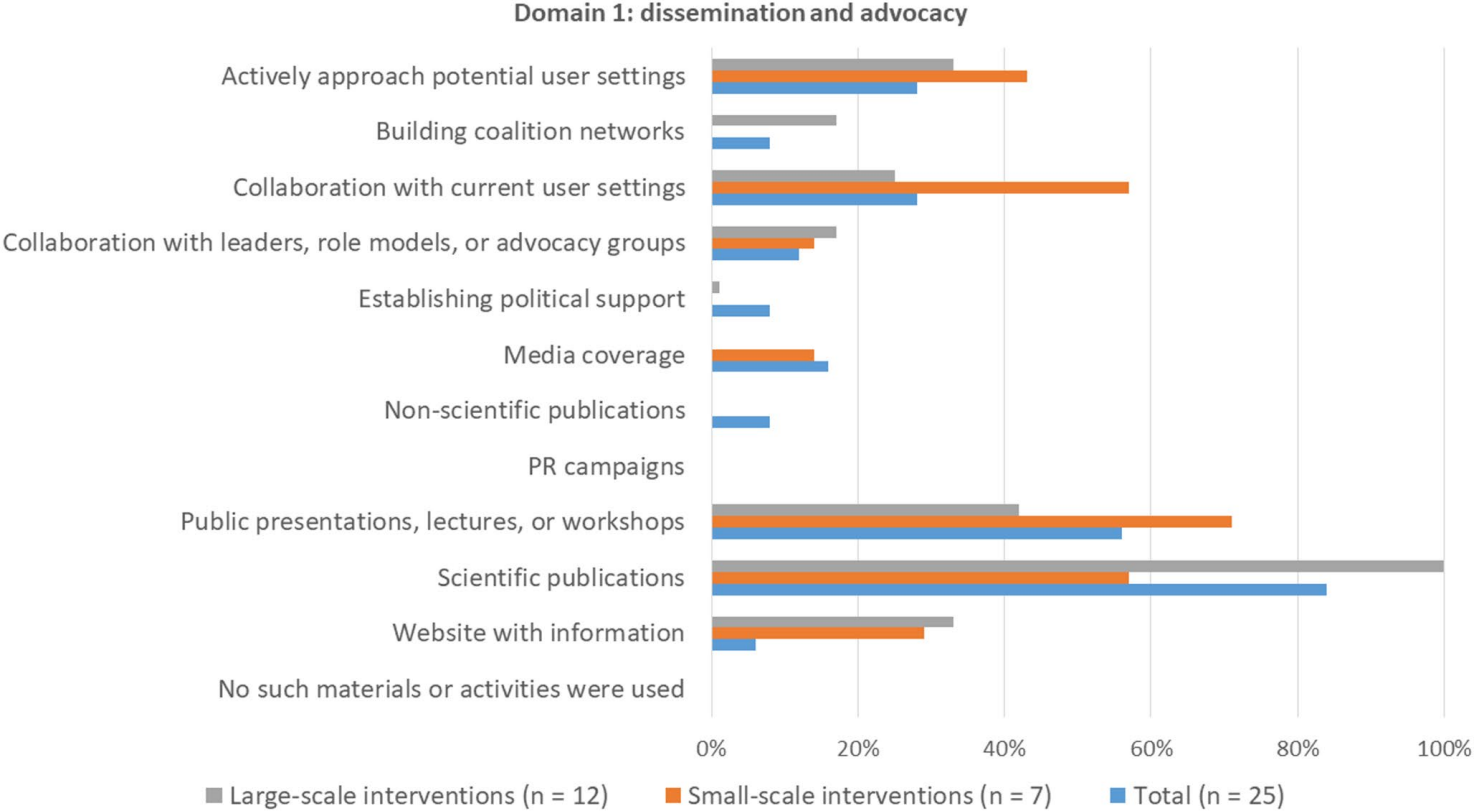
Frequencies of application of strategies for dissemination and advocacy. Participants could select up to three strategies that were most relevant for scale-up. Percentages represent the number of times a strategy was selected as one of the most relevant strategies

**Fig. 4:**
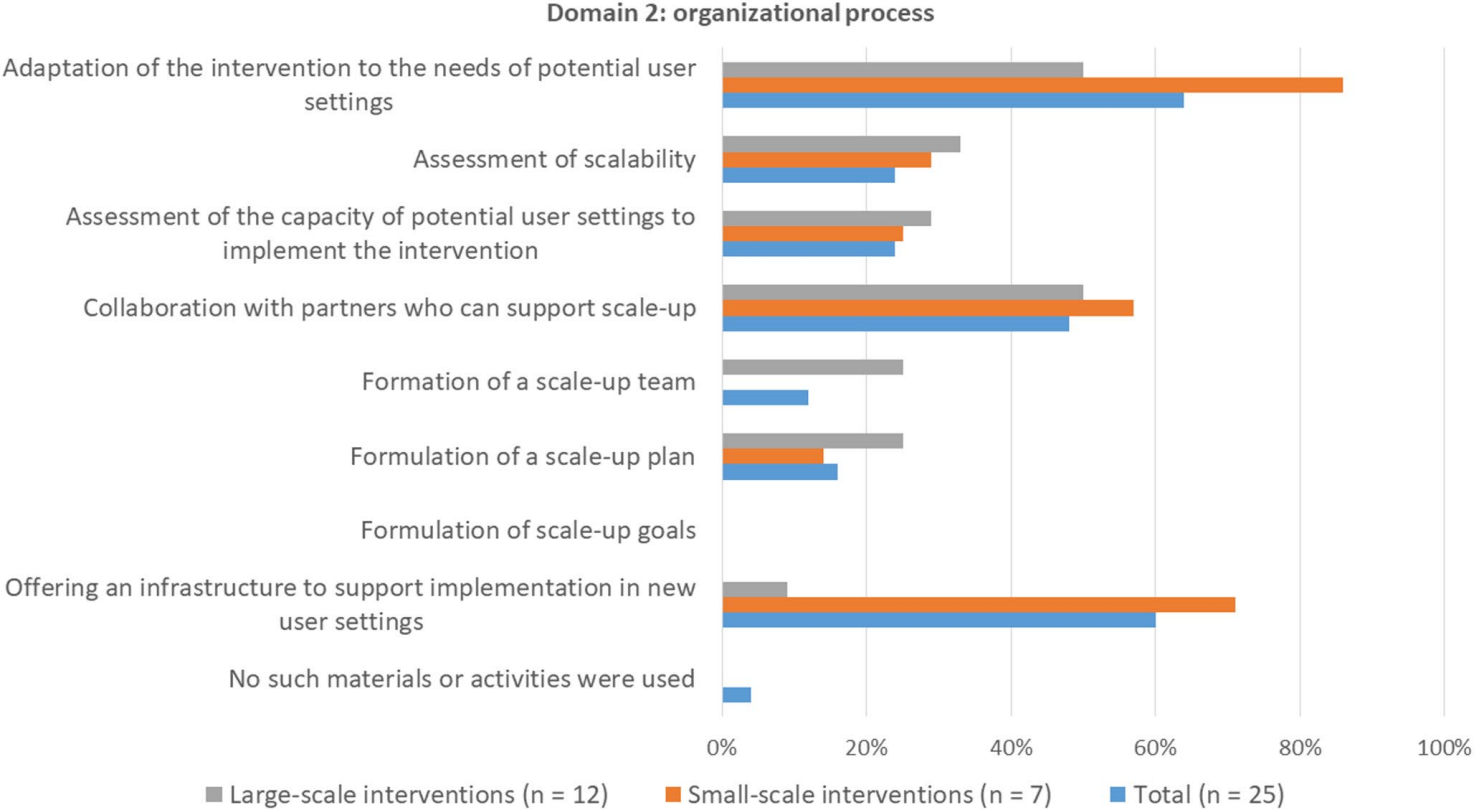
Frequencies of application of strategies for organizational process. Participants could select up to three strategies that were most relevant for scale-up. Percentages represent the number of times a strategy was selected as one of the most relevant strategies

**Fig. 5:**
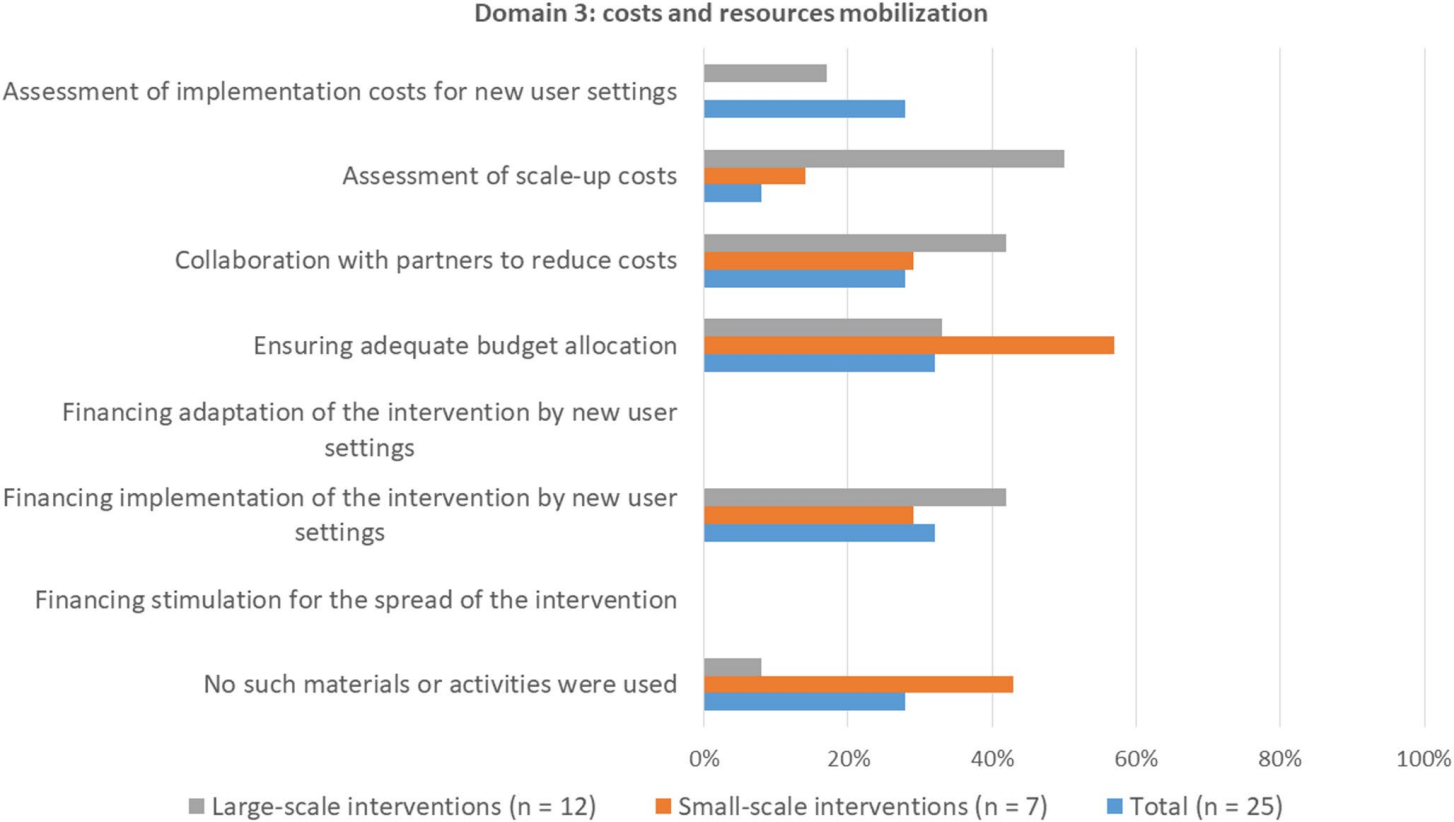
Frequencies of application of strategies for mobilizing costs and resources

**Fig. 6:**
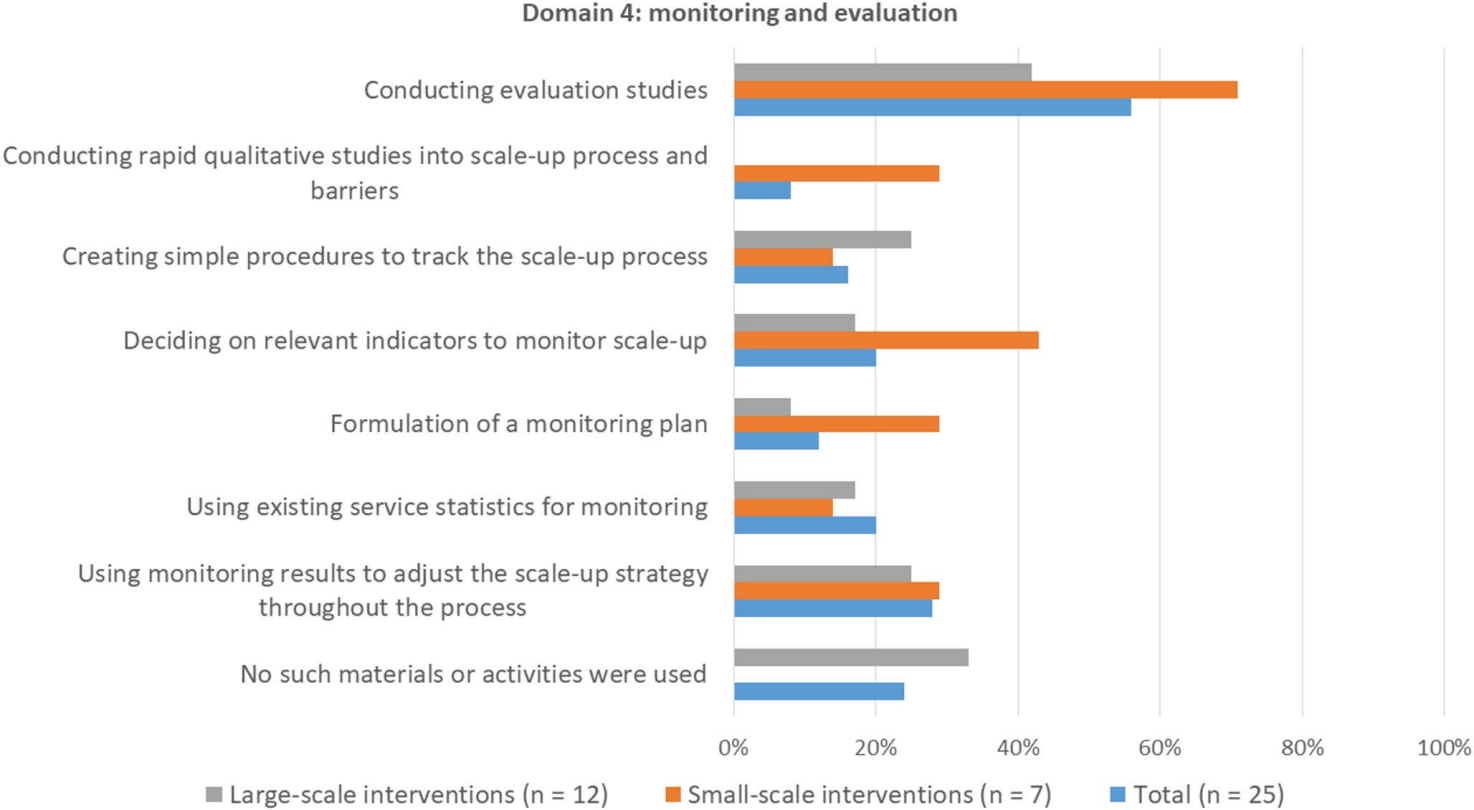
Frequencies of application of strategies for monitoring and evaluation. Participants could select up to three strategies that were most relevant for scale-up. Percentages represent the number of times a strategy was selected as one of the most relevant strategies

Differences in applied strategies between large- and small-scale interventions are presented per domain of scale-up strategies in Figs. 3, 4, 5 and 6. Likewise, differences between in-progress examples and scaled-examples are presented based on the interview data. Additional quotes can be found in Additional File 6.

#### Domain 1: Strategies for dissemination and advocacy

All survey participants indicated that strategies to disseminate and advocate for scale up were used (Fig. 3). The most frequently used strategies for dissemination were scientific publications (84%) and public presentations, lectures, or workshop (56%). The strategies ‘building coalition networks’ and ‘scientific publications’ were applied more often for large-scale interventions compared to small-scale interventions.

For the scaled interventions reported on in the interviews, strategies for dissemination and advocacy were tailoring communication about the intervention to setting goals, spreading through existing networks, scientific dissemination, and inclusion in intervention registries. Spreading through existing networks and scientific dissemination were considered important strategies for the scaled- and in-progress examples. However, inclusion in intervention registries was only mentioned for scaled-examples, for all of which it was considered one of the most helpful strategies for dissemination.

*“[Registration in intervention registries] allowed the program to scale across the country in a type of designation that was critical for it.”* – Scaled-example 1, North America.

Additionally, tailoring the framing of the intervention to align with the goals of the target setting was only mentioned by one of the scaled-examples. This was described as a helpful strategy for dissemination.

*“I had to learn to understand the culture of the school […]*,* and every school has a mission statement. I go back and look at those […] and I try to pitch to whatever their need is and work with them.”* – Scaled-example 3, North America.

#### Domain 2: Strategies for organizational process

Most survey participants indicated that they applied strategies to organise the scale-up of their intervention (Fig. 4). Adaptation of the intervention to the needs of potential user settings (64%) and offering an infrastructure to support implementation in new user settings (60%) were applied for most for both small- and large scale interventions. For large-scale interventions, the formation of a scale-up team and scale-up plan were applied more often as a strategy to undertake scale-up than for small-scale interventions.

Most of the strategies for the organizational process of scale up that were mentioned for the scaled-examples, were also applied for the in-progress examples. Adapting the intervention was mentioned for three in-progress examples, but one mentioned it as a strategy for sustainment within an existing setting rather than a scale-up strategy, and the other two were still preparing for scale-up. Moreover, for all three scaled-examples, adaptations were highlighted as an important strategy for dealing with scale-up barriers, such as shortening the intervention or delivering it online.

*“Part of the challenges of scaling the secondary [school] program led us to design the elementary [school] program. That’s a lot easier to deliver.”* – Scaled-example 1, North America.

While an intervention core team was mentioned as a strategy by two in-progress examples, this was also considered an important strategy by all three scaled-examples. For two examples, it entailed having a group of certified trainers around the world who delivered train-the-trainer sessions in new intervention settings. For the other, it was a national intervention team which provided train-the-trainer sessions and coordinated the implementation and scale-up of the intervention.

*“I support all our secondary [school] implementation here in the United States […] and we have our national staff of trainers that train across the country.”* – Scaled-example 1, North America.

Additionally, two scaled-examples had local teams involved in intervention implementation, and they provided local train-the-trainer sessions to further disseminate the intervention within smaller regions.

*“We run train-the-trainers for training local trainers*,* either educators or US district level employees*,* and we have a second layer called district service centres that support like 20 districts of schools and they have trained professionals to do training.”* – Scaled-example 1, North America.

Furthermore, only the three scaled-examples talked about train-the-trainer sessions as an organizational strategy, whereas others only provided basic deliverer trainings or no trainings at all. One scaled-example additionally provided all-staff training for intervention schools, where deliverers learned to talk about suicidality, and webinars for intervention deliverers across the country.

*“Once schools have bought in or have funding*,* they get trained their entire staff*,* so that all adults in the building have the language to talk about [suicidality]. […] We offer some national community learning for trainers [and intervention providers] through webinars.”* – Scaled-example 1, North America.

#### Domain 3: Strategies for mobilizing costs and resources

Most survey participants indicated that they applied strategies to mobilize costs and resources for the scale-up of their intervention (Fig. 5). Assessment of scale-up costs (50%) was applied most often for large-scale interventions. Assessment of both implementation costs for new user settings and scale-up costs, collaboration with partners to reduce costs, and financing implementation of the intervention by new user settings were applied more often for large-scale interventions compared to small-scale interventions. For small-scale interventions, it was more common that no strategies for mobilizing costs and resources were used.

Participants could select up to three strategies that were most relevant for scale-up. Percentages represent the number of times a strategy was selected as one of the most relevant strategies.

In the interviews, the following financing strategies were mentioned for the scaled-examples: reducing implementation costs for new user settings, applying for research and/or training funds, commercializing the intervention, and arranging subsidy for new user settings. No notable differences between the scaled-examples and in-progress examples were found in terms of what financial strategies they applied.

#### Domain 4: Strategies for evaluation and monitoring

Most survey participants reported that they applied strategies to monitor and report on the scale-up of their intervention (Fig. 6). The most frequently applied strategy for both scaled-and in-progress examples was conducting an evaluation (56%). Procedures for tracking the scale-up process were applied more often for large-scale intervention than small-scale interventions. Monitoring strategies were applied for all small-scale interventions, while no such strategies were applied for 33% of the large-scale interventions.

The following strategies for evaluation and monitoring were mentioned in the interviews about the scaled-examples: effect and process evaluations, developing evaluation tools, monitoring fidelity, and tracking systems. Of these, only process evaluations were also applied for in-progress examples. For all three scaled-examples, monitoring fidelity was discussed as an important strategy for monitoring scale-up. This was done in various ways, such as observing intervention delivery, mock-sessions, and analysing video-recordings of intervention delivery by newly trained deliverers.

*“The first two times they train in school*,* we train alongside them so we can monitor fidelity and quality.”* – Scaled-example 1, North America.

Responses with missing data were excluded if the participant only completed the section relating to the ‘intervention’ and ‘participant characteristics’. Survey data were analysed by calculating descriptive statistics in SPSS. To differentiate between the level of scale-up, we categorised interventions as scaled up on either a large scale (implemented in > 10 schools) or small-scale (implemented in 1–10 schools). As there is no clear cut-off point for when an intervention can be considered to be implemented at scale, we chose 10 schools as a cut-off point as this led to a roughly equal distributions of interventions across the categories small and large scale.

Interview transcripts were analysed thematically in Atlas.ti. Thematic analysis was used to identify recurring themes related to scale-up in school settings. First, eight transcripts were open-coded by three researchers (KJ, RS, DP) to ensure triangulation. These researchers then categorized their codes through axial coding, and a code tree was developed in agreement between all three researchers (Additional File 4). Main themes identified were based on the scale-up framework from Simmons and Shiffman (2007): “scale-up barriers,” “scale-up facilitators,” “strategies for dissemination and advocacy,” “strategies for organizational processes,” “strategies for mobilizing costs and resources,” and “strategies for evaluation and monitoring.” Next, the lead author (KJ) coded all transcripts deductively using the code tree. Three researchers (KJ, FN, SR) reviewed the analyses and regrouped codes into three themes, with corresponding subthemes: (1) decision to scale-up, (2) scale-up determinants (i.e. intervention characteristics, resource availability, and school context), and (3) scale-up strategies (i.e. dissemination and advocacy, organizational process, mobilizing costs and resources, and evaluation).

To explore findings in relation to scale-up success, interventions were categorized by three researchers (KJ, FN, SR) as: (1) scaled-up (i.e., efforts to scale-up were ongoing, and > 10 new settings implemented the intervention after the trial), (2) in-progress (i.e., ongoing projects with intention to scale-up, but ≤ 10 settings implemented the intervention after the trial), (3) discontinued interventions (i.e., interventions no longer implemented). The lead author (KJ) further analysed the data within each subtheme. In case of discrepancies during the analysis process, regular discussions were held between all researchers until consensus was reached.

## Discussion

In this study, we explored the scaling up of school-based interventions for preventing anxiety, depression, and suicidality in youth. We found that for about half of interventions included in the survey there was an intention to scale-up, and only three interventions included in the interviews were implemented at a large scale (> 10 schools). Determinants for scale-up of the included interventions related to the intervention itself, resources availability, and school context. We found differences between small-scale and large-scale interventions in the use of scale up strategies, which we explore further below.

### Extent to which interventions are scaled-up

Despite the need to scale-up school-based anxiety, depression, and suicidality prevention interventions, our findings suggest that few evidence-based interventions are implemented at a large scale after their initial trial period. In congruence with other studies into determinants of scale-up (Fagan et al. 2019; Milat et al. 2015; Smit et al. 2024a, b; Troup et al. 2021), availability of funding was a frequently mentioned factor. However, irrespective of demonstrable intervention effectiveness, funds are often only provided for the development and testing of an intervention, and not for its scale-up (Ramani-Chander et al., 2023). This highlights an important gap between what is needed in practice and what is being funded.

Lack of funding is not the only issue for limited scale-up. Successfully scaling an intervention also depends on its scalability; that is, its capacity to expand coverage to a larger share of the target population without losing effectiveness (Milat et al., 2013). In this study, nearly half of the included school-based mental health interventions were discontinued after their trial phase, which may indicate that many were not originally designed for scalability. This suggests that incorporating future scale-up potential during the intervention development phase is needed (World Health Organization, 2011; Zamboni et al., 2019). Similarly, evaluating scalability can assist policymakers and practitioners in selecting interventions with a higher likelihood of being scaled successfully (Milat et al., 2020).

### Strategies for successful scale-up

In the interviews, intervention registries were described by participants as a helpful strategy for intervention dissemination. This is consistent with Axford and colleagues (2022), who describe that such registries support decision-makers in identifying and selecting suitable interventions. Potentially, including an intervention in intervention registries increases the legitimacy and trustworthiness of the intervention, which subsequently facilitates potential scale-up. While scientific publications were frequently mentioned, tailored communication and spreading through existing networks were consistently described as important strategies, in line with results from others (Kwan et al., 2022; Tenney et al., 2022).

Within the organizational process domain, both survey and interview participants frequently mentioned adapting the intervention to potential user settings as an important strategy for scale-up. It is suggested that increased intervention adaptability contributes to scale-up success, mainly by improving equity and reaching diverse and disadvantaged target groups (Koorts et al., 2025). Adaptation is often necessary to improve the fit and potential adoption into new user settings, but also for sustaining interventions as implementation contexts change over time (La Bash et al., 2019). However, as higher levels of fidelity are related to better intervention outcomes (Durlak & DuPre, 2008), balancing fidelity and adaptation is warranted. This is supported by our finding that monitoring fidelity was a valuable evaluation strategy for all three scaled-examples. However, that requires identification of the core components of an intervention, and knowledge of where there is room for change (Toomey et al., 2020).

For many interventions included in our study, an important barrier for scale-up were financial constraints. We found that strategies for cost assessment and cost reduction were applied more often for large-scale interventions compared to small-scale interventions. It is unclear from our findings whether this is only because larger scale interventions required more financing, or if the lack of financial support prevented further intervention expansion. Milat and colleagues (2015) did find that financial strategies are key success factors for scale-up, stressing the need to mobilize costs and resources when scaling up.

Comparing our results to what is known about implementation strategies, we identified both overlap and distinctions. Waltz et al. (2015) clustered the ERIC (Expert Recommendations for Implementing Change) strategies compiled by Powell et al. (2015) into nine groups. Nathan et al. (2022) adapted the ERIC implementation strategies to focus more explicitly on sustainment beyond initial implementation. For scale-up, however, a compilation of strategies has not been established thus far. There is some overlap in the strategies we found compared to the ERIC implementation strategies, such as in the category of strategies “adapt and tailor to context”. However, the underlying purpose differs: as an implementation strategy, adaptation aims to improve fit and integration within a specific setting. In the context of our current study, we found that as a scale-up strategy, adaptation focused on enabling reach across diverse settings. For instance, one scaled-example modified its delivery approach to target primary schools in order to expand its target population. Financial strategies illustrate a similar pattern. For implementation, financial planning typically involves securing resources to introduce the intervention in a single setting (Powell et al., 2015). In contrast, scale-up requires funding mechanisms that can sustain implementation across multiple settings, at a much larger scale. These examples highlight that while implementation and scale-up share strategic categories, their objectives and operationalization differ. Implementation strategies prioritize depth – ensuring successful adoption within a specific context – whereas scale-up strategies prioritize breadth – extending reach across varied contexts. Recognizing these distinctions is necessary for designing effective approaches that move beyond initial adoption toward sustainable, widespread impact.

### Strengths and limitations

Several strengths and limitations to our study should be noted. Our sequential mixed methods design helped us gain both a broad and in-depth insight into the scale-up processes: the surveys provided a wide reach of school-based mental health interventions, while the additional interviews enabled us to gain a deeper understanding of unique case examples. Although these examples provide valuable insights into determinants and strategies that contributed to the scale-up school-based mental health interventions, it should be noted that these examples are only illustrative and do not provide evidence for best practice. As this is a descriptive study, we were not able to estimate the effectiveness of the scale-up strategies for these interventions. Currently, little is known about the effectiveness of such scale-up strategies (Alonge et al., 2020; Ben Charif et al., 2017). As a result, providing more guidance is complicated. Our findings suggest some starting points for successful scale-up. However, further research into the effectiveness of scale-up strategies is needed. Nevertheless, our interview data demonstrated the relevance and utility of several scale-up strategies for the case examples we discussed. While our study design did not allow us to analyse the effectiveness of scale-up strategies, we were able to identify several strategies that were considered helpful for scaled-up school-based interventions for anxiety, depression, and suicidality prevention.

We recognize that the number of participants in this study is limited, and that we were able to interview only three participants about examples of scaled interventions. In part, this may be due to the fact that so few interventions reach scale-up, and thus the potential participants for recruitment are reduced. Also, there are often only specific individuals with sufficient knowledge of the scale up process (Koorts et al., 2021). Given the small pool of participants, it does mean that we may have missed other determinants and strategies for scale-up. Furthermore, all scaled-examples were interventions developed and (mostly) implemented in North America. We therefore do not know how generalizable the determinants and strategies we found are to other countries.

There is also the potential for self-selection bias in our sample, as authors with more successful scale-up stories may be more likely to participate in the study. However, as there were also a number of participants who reported on discontinued intervention, we believe this was not the case.

## Conclusion

Our analysis of real-world scale-up processes revealed that only a small number of school-based mental health interventions are implemented at scale. Our findings suggest that funding for the scale-up of effective interventions, developing scalable interventions, and assessing scalability when making scale-up decisions may contribute to scale-up. Although our results do not allow us to conclude which scale-up strategies are successful, they provide illustrative examples that may help advance the field of scale-up research. We were able to identify several strategies that were considered helpful for three examples of successfully scaled-up school-based interventions for anxiety, depression, and suicidality prevention. Their experiences may offer guidance for future scale-up of mental health prevention interventions, and within other fields.

## Supplementary Information

Below is the link to the electronic supplementary material.

Supplementary Material 1

Supplementary Material 2

Supplementary Material 3

Supplementary Material 4

Supplementary Material 5

Supplementary Material 6

## Data Availability

Quantitative data is provided within the manuscript and supplementary information files.
