# Supplementary material for: Analysis of real-world scale-up processes for school-based mental health interventions
Source: Adm Policy Ment Health. 2026 Mar 9;53(3):224–39. doi: 10.1007/s10488-026-01491-0 (PMC13221318; doi:10.1007/s10488-026-01491-0)
Supplement: Supplementary file 1 — Supplementary Material 1 [file 10488_2026_1491_MOESM1_ESM.pdf]

A Checklist for mixed methods research Manuscript Preparation and Review, Lee et al. (2022)

		On page(s)
Rational and description of MMR design	Provide a clear statement of the study purpose	4
	Explicitly describe the MMR design in accordance with Creswell's (2015) typology and use a diagram to illustrate the relationship and sequence of qualitative and quantitative research components	4-7
	Justify why the MMR design is appropriate for meeting the study purpose	4
Transpareny in describing method details	Describe the study population(s) and samples (s, e.g. who, what, how many)	5
	Describe the sampling procedures (including inclusion and exclusion criteria, recruitment)	5
	Describe qualitative data collection processes (how often data were collected, who collected the data, what kind of data collection instruments were used, how data were recorded - e.g. notes, transcripts)	6-7
	Describe quantitative data collection processes (how often data were collected, who collected the data, what kind of data collection instruments were used measurements, validity/reliability)	5-6

	Describe qualitative data analysis processes (coding, single or multiple coders, replication logic, credibility)	6-7
	Describe quantitative data analysis procedures (missing data and how they are handled, statistical tests used)	5-6
Integration of qualitative and quantitative research components	Interpret qualitative analysis results with appropriate quotes if necessary	7-18
	Interpret quantitative analysis results in consideration of statistical significance, selection bias, and threats to validity	7-18
	Compare qualitative and quantitative results	7-18
	Address divergencies and inconsistencies between qualitative and quantitative results	7-18
