# Supplementary material for: Analysis of real-world scale-up processes for school-based mental health interventions
Source: Adm Policy Ment Health. 2026 Mar 9;53(3):224–39. doi: 10.1007/s10488-026-01491-0 (PMC13221318; doi:10.1007/s10488-026-01491-0)
Supplement: Supplementary file 2 — Supplementary Material 2 [file 10488_2026_1491_MOESM2_ESM.docx]

Standard: Introduction (1 Question)

Standard: Part 1 (5 Questions)

Block: Start Part 2 (4 Questions)

Branch: New Branch

If

If Do you still work in this sector? No Is Selected

Block: Q8a (1 Question)

Standard: Q9 (1 Question)

Standard: Q10 (1 Question)

Standard: Q11 (1 Question)

Standard: Q12 (1 Question)

Branch: New Branch

If

If Is ${Q66/ChoiceTextEntryValue} an ongoing project? Yes Is Selected

Block: Q12a (1 Question)

Standard: Introduction part 3 (1 Question)

Branch: New Branch

If

If Part 3 focuses on the process after the trial. We are interested in any attempts to scale up the... Yes Is Selected

Block: Q14 scale up (1 Question)

Block: Q15 scale up (1 Question)

Block: Q16 scale up (1 Question)

Block: Q17 scale up (1 Question)

Block: Q18 scale up (1 Question)

Block: Q19 scale up (1 Question)

Branch: New Branch

If

If QID33 q://QID33/SelectableChoice/2 Is Selected

Branch: New Branch

If

If Part 3 focuses on the process after the trial. We are interested in any attempts to scale up the... No Is Selected

Block: Q20+Q21 no scale up (2 Questions)

Standard: Comments (1 Question)

Standard: Interview yes/no (1 Question)

Branch: New Branch

If

If Would you like to participate in an online interview to further elaborate on your answers? Throug... Yes Is Selected

Block: Contact details (1 Question)

| Page Break |  |
| --- | --- |

Start of Block: Introduction

Consent Thank you for participating in this study. The aim of this study is to gain insight into scale up processes of school-based mental health interventions after their initial trial period. We are interested to know if and how the intervention of the trial we refer to is being or has been scaled up. We believe your experiences are valuable to inform future scale up projects. Therefore, we invite you to fill in this short survey.

 It should take about 5 minutes to complete the survey. If you would like more information about the study, please read the Plain Language Statement

 **Procedure**
 Please read the questions carefully and answer as honestly as possible by selecting the most suitable option. When you are asked to write an answer, please do so the best you can in the space provided.

 **Consent**
 1. By clicking ‘I Agree’ below you are giving consent for your answers to be used as part of this research.
 2. You are also confirming that you have had a chance to read the Plain Language Statement above and have had the opportunity to ask further questions or seek clarification about this research.
 3. The researchers have agreed not to reveal your identity and personal details.
 4. If you require further information or if you have any questions or problems concerning this project, please contact …

 If you wish to take part in this survey, please click 'I Agree' to begin. 
 *If you do not agree, please close the questionnaire.*

- I Agree (1)

End of Block: Introduction

Start of Block: Part 1

Q0 Part 1 is about the intervention or program of the paper we are referring to (you can find this in the invitation email).

Please provide a short name for the intervention/program/approach of the paper we are referring to. This data will not be saved, but will be used to tailor the following questions.

________________________________________________________________

| Page Break |  |
| --- | --- |

Q1 When was ${Q0/ChoiceTextEntryValue} first applied in practice? (Please indicate or estimate the year and month)

________________________________________________________________

Q2 By estimation, how many schools have implemented ${Q0/ChoiceTextEntryValue} so far?

________________________________________________________________

| Page Break |  |
| --- | --- |

Q3 What is the focus of the intervention?

- Anxiety (1)
- Depression (2)
- General mental health (3)
- Suicide (4)
- The intervention focuses on multiple aspects, namely: (5) __________________________________________________
- Other, please specify: (6) __________________________________________________

Q4 What is the target population of the intervention?

- Primary school students (1)
- Secondary school students (2)
- Vocational school students (3)
- University students (4)
- The intervention is offered to multiple populations, namely: (5) __________________________________________________
- Other, please specify: (6) __________________________________________________

End of Block: Part 1

Start of Block: Start Part 2

Q5 Part 2 is about your personal characteristics.

 In which country do you primarily work?
 *Please note, if you regularly work in more than one country please select where you spend the majority of your time.*

________________________________________________________________

| Page Break |  |
| --- | --- |

Q6 Please select your age range:

- 18-24 years (1)
- 25-29 years (2)
- 30-34 years (3)
- 35-39 years (4)
- 40-44 years (5)
- 45-49 years (6)
- 50-54 years (7)
- 55-59 years (8)
- 60+ years (9)

| Page Break |  |
| --- | --- |

Q7 In which sector did you work during trial period of ${Q0/ChoiceTextEntryValue}?
 *Please select all that apply.*

- University/academic position (1)
- Mental health care (2)
- Other health care sector (3)
- Non-governmental Organization (NGO) or Non-profit organization (4)
- International mental health organization (not involving patient contact) (5)
- National mental health organization (not involving patient contact) (6)
- Community mental health organization (not involving patient contact) (7)
- Research consultancy (8)
- Other, please specify (9) __________________________________________________

Q8 Do you still work in this sector?

- No (1)
- Yes (2)

End of Block: Start Part 2

Start of Block: Q8a

Q8a In which sector do you currently work?
 *Please select all that apply.*

- University/academic position (1)
- Mental health care (2)
- Other health care sector (3)
- Non-governmental Organization (NGO) or Non-profit organization (4)
- International mental health organization (e.g. an association, an alliance) (5)
- National mental health organization (6)
- Community mental health organization (7)
- Research consultancy (8)
- Other, please specify (9) __________________________________________________

End of Block: Q8a

Start of Block: Q9

Q9 How long have you been working in this sector?

- 1-5 years (1)
- 6-10 years (2)
- 11-15 years (3)
- 16-20 years (4)
- 21-25 years (5)
- Over 25 years (6)

End of Block: Q9

Start of Block: Q10

Q10 What is your current position?

- Academic (research, teaching, and/or lecturing) (1)
- Mental healthcare professional (2)
- Consultant (3)
- Policy maker/policy officer (4)
- Senior manager/CEO of organization (5)
- Student (including higher degree) (6)
- Industry professional (7)
- Other, please specify (8) __________________________________________________

End of Block: Q10

Start of Block: Q11

Q11 What is or was your role in ${Q0/ChoiceTextEntryValue}?

- Principal investigator (1)
- Project manager (2)
- Co-investigator (3)
- PhD student (4)
- PhD supervisor (5)
- Clinician (6)
- Other, please specify (7) __________________________________________________

End of Block: Q11

Start of Block: Q12

Q12 Is ${Q0/ChoiceTextEntryValue} an ongoing project?

- No (1)
- Yes (2)

End of Block: Q12

Start of Block: Q12a

Q12a Are you still involved in ${Q0/ChoiceTextEntryValue}?

- No (1)
- Yes (2)

End of Block: Q12a

Start of Block: Introduction part 3

Q13 Part 3 focuses on the process after the trial. We are interested in any attempts to scale up the intervention after the trial. In this survey, we define scale up as deliberate efforts to increase the impact of an intervention so that more people can benefit from the intervention.

Was ${Q0/ChoiceTextEntryValue} scaled up / is ${Q0/ChoiceTextEntryValue} being scaled up in practice after the trial?

- No (1)
- Yes (2)

End of Block: Introduction part 3

Start of Block: Q14 scale up

Q14 What led to the decision to scale up ${Q0/ChoiceTextEntryValue} after the trial period?
 *You can select multiple answers.*

- Accreditation or certification of the intervention (10)
- Availability of financial resources for scale up (1)
- Commitment from the intervention developers/project team (2)
- Interest among stakeholders to implement the intervention (3)
- Intervention addresses a key need in the community (4)
- Intervention was found to be effective (5)
- Political will and support to scale up (7)
- I am not sure (8)
- Other, please specify: (9) __________________________________________________

End of Block: Q14 scale up

Start of Block: Q15 scale up

| 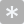 |
| --- |

Q15 What materials or activities were used after the trial to disseminate (spread) and advocate for ${Q0/ChoiceTextEntryValue}?
*In case more than three of the materials or activities below were used, please select the three that were most important.*

- Actively approaching (potential) user settings (1)
- Building coalition networks (2)
- Collaboration with leaders, role models or advocacy groups (3)
- Collaboration with current user settings (4)
- Establishing political support (5)
- Media coverage (6)
- Non-scientific publications (7)
- PR campaign(s) (8)
- Public presentation, lectures, workshops (9)
- Scientific publications (10)
- Website with information (11)
- None such materials and activities were used (14)
- Other, please specify: (15) __________________________________________________

End of Block: Q15 scale up

Start of Block: Q16 scale up

| 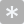 |
| --- |

Q16 What materials or activities were used after the trial to organize the scale up of ${Q0/ChoiceTextEntryValue}?
 *In case more than three of the materials or activities below were used, please select the three that were most important.*

- Assessment of scalability of the intervention (1)
- Assessment of capacity of (potential) user settings to implement the intervention (2)
- Adapting the intervention to the needs of (potential) user settings (3)
- Collaborating with partners who can support scale up (4)
- Formation of a scale up team (5)
- Formulating a scale up plan (6)
- Formulating scale up goals (7)
- Offering infrastructure to support implementation (e.g. toolkits, training, support) (8)
- No such materials or activities were used (9)
- Other, please specify: (10) __________________________________________________

End of Block: Q16 scale up

Start of Block: Q17 scale up

| 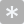 |
| --- |

Q17 What materials or activities were used after the trial to mobilize costs and resources for the scale up of ${Q0/ChoiceTextEntryValue}?
 *In case more than three of the materials or activities below were used, please select the three that were most important.*

- Assessment of scale up costs (1)
- Assessment of implementation costs for new user settings (2)
- Collaboration with partners to reduce costs (3)
- Ensuring adequate budget allocation (4)
- Financing adaption of the intervention by new user settings (5)
- Financing implementation of the intervention in new user settings (6)
- Financing stimulation for spread of the intervention (7)
- No such material and activities were used (8)
- Other, please specify: (9) __________________________________________________

End of Block: Q17 scale up

Start of Block: Q18 scale up

| 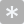 |
| --- |

Q18 What materials or activities were used after the trial to monitor the scale up of ${Q0/ChoiceTextEntryValue}?
 *In case more than three of the materials or activities below were used, please select the three that were most important.*

- Conducting evaluation studies (1)
- Conducting rapid qualitative studies to gain insight into the process and barriers to scale up (2)
- Creating simple procedures for tracking the scale up process (3)
- Decide on relevant indicators to monitor scale up (4)
- Formulating a monitoring plan (5)
- Using existing service statistics for monitoring (6)
- Using monitoring results to adjust the scale up strategy throughout the process (7)
- No such materials and activities were used (8)
- Other, please specify: (9) __________________________________________________

End of Block: Q18 scale up

Start of Block: Q19 scale up

Q19 To what scale was ${Q0/ChoiceTextEntryValue} scaled up or to what scale will the intervention be scaled up?

- Local scale (1)
- Regional scale (2)
- National scale (3)
- International scale (4)
- Other, please specify: (5) __________________________________________________

End of Block: Q19 scale up

Start of Block: Q20+Q21 no scale up

Q20 Was there an interest or intention to scale up ${Q0/ChoiceTextEntryValue} (even though it was not actually scaled up)?
 *You can select multiple answers.*

- No (1)
- Yes, from intervention developers (2)
- Yes, from the research team (3)
- Yes, from the target population/target setting (4)
- Yes, from governmental parties (5)
- Yes, from others, please specify: (6) __________________________________________________
- I am not sure (7)

| Page Break |  |
| --- | --- |

Q21 What led to the decision to not scale up after the trial period, e.g. why ${Q0/ChoiceTextEntryValue} stopped after the trial?
 *You can select multiple answers.*

- No accreditation or certification of the intervention (10)
- Lack of financial resources for scale up (1)
- No commitment from the intervention developers/project team (2)
- Lack of interest among stakeholders to implement the intervention (3)
- Intervention does not address a key need in the community (4)
- Intervention was not found to be effective (5)
- No political will and support to scale up (7)
- I am not sure (8)
- Other, please specify: (9) __________________________________________________

End of Block: Q20+Q21 no scale up

Start of Block: Comments

Q22 Do you have any comments or suggestions you think are important to mention?

________________________________________________________________

End of Block: Comments

Start of Block: Interview yes/no

Q23 Would you like to participate in an online interview to further elaborate on your answers? Through these interviews, we want to gain more in-depth insight into what happened with an intervention after the initial trial period. The interview will be approximately one hour.

We are also interested in hearing more from you if the intervention was not scaled up.

- Yes (1)
- No (3)

End of Block: Interview yes/no

Start of Block: Contact details

Q24 Please provide your name and email address so we can contact you for participation in an interview.
 *Your contact details will be stored separately from the other answers in the survey. Your answers cannot be linked to your personal information, your answers remain anonymous.*

- Name (4) __________________________________________________
- Email (5) __________________________________________________

End of Block: Contact details
