# Supplementary material for: Analysis of real-world scale-up processes for school-based mental health interventions
Source: Adm Policy Ment Health. 2026 Mar 9;53(3):224–39. doi: 10.1007/s10488-026-01491-0 (PMC13221318; doi:10.1007/s10488-026-01491-0)
Supplement: Supplementary file 3 — Supplementary Material 3 [file 10488_2026_1491_MOESM3_ESM.docx]

**Study 2 Scale up Strategies: topic lists (English)**

***If no attempts were made to scale up the intervention***

| **Introduction:**   - Goal interview:   - Gain insight into factors that prompt scale up decisions for school-based mental health interventions   - Gain insight into scale up processes   - Learn from practice for future scale up projects - We will be talking about what happened to a school-based mental health intervention in which you are or were involved after its trial period ended - Questions? - Permission to record | |
| --- | --- |
| **Topic** | **Questions** |
| **Participant & intervention** | Could you please introduce yourself?  Could you provide a short description of the intervention?   - How was the intervention developed?   - By whom? - What is the target population? - Who was involved in the delivery of the intervention? - What other stakeholders were involved in the intervention? - In which setting was the intervention delivered? |
| **Scale up decisions** | What happened after the intervention trial?  Was there an interest to scale up the intervention after the trial?   - If there was:   - Who were interested?   - Why were they interested? - If there was not:   - Why not?   What were reasons that the scale up did not happen?   - Was there a decision to not scale up? - What led to this decision?   What factors hindered the scale up of the intervention?  *(example: not effective, financing, no support, no time and/or staff available, etc.)* |
| **Closing** | Anything to add/questions?  Thank you  Explain interview summary  Stop recording |

***If any scale up attempts were made***

| **Introduction:**   - Goal interview:   - Gain insight into factors that prompt scale up decisions for school-based mental health interventions   - Gain insight into scale up processes   - Learn from practice for future scale up projects - More information:   - We will be talking about the scale up of a school-based mental health intervention in which you are or were involved.   - By scale up we mean deliberate actions to enlarge the population that is reached by an intervention. So it includes spreading the intervention to new settings, supporting new settings who implement the intervention, arranging resources for scaling up and monitoring the process.   - Scale up is sometimes confused with implementation. By implementation we mean deliberate actions to put an intervention to practice within a new setting. In this interview, we will mostly talk about scale up. - Questions? - Permission to record | |
| --- | --- |
| **Topic** | **Questions** |
| **Participant & intervention** | Could you please introduce yourself?  Could you provide a short description of the intervention?   - How was the intervention developed?   - By whom? - What is the target population? - Who was involved in the delivery of the intervention? - What other stakeholders were involved in the intervention? - In which setting was the intervention delivered? |
| **After trial** | What happened to the intervention after the trial period  Which factors helped to boost the scale up of the intervention?  *(example: effectiveness, financing, support, time and/or staff available, etc.)*  What factors limited the scale up of the intervention?  *(example: financing, no support, no time and/or staff available, etc.)* |
| **Scale up decisions** | To what extent was the scale up planned?  What led to the decision to scale up the intervention?  Who were involved in this decision?  Were any scale up goals formulated at any point?   - What are they? |
| **Scale up process** | *When scaling up, different kinds of actions can be taken. We differentiate between:*   - *Actions to spread the intervention to new settings* - *Actions to support new settings who want to implement the intervention* - *Actions to finance the scale up* - *Actions to monitor the scale up process*   How do new settings know about the intervention and how to get started?   - Who were involved? - How effective was this approach? - What worked best in disseminating the intervention? - What would you do different in future scale up projects?   *(Examples: establishing support, media campaigns, actively approaching new settings, etc.)*  How is the practical side of supporting new settings organized?   - Who were involved? - How effective was this approach? - What worked best in organising the scale up of the intervention? - What would you do different in future scale up projects?   *(Examples: adapting the intervention to meet practical needs, offering training and support, etc.)*  How was the scale up financed?   - Who were involved? - How effective was this approach? - What worked best in mobilizing costs and resources? - What would you do different in future scale up projects?   *(Examples: budget allocation, applying for funds, assessment of scale up costs)*  How is or was the use of the intervention in new settings monitored?   - Who were involved? - How effective was this approach? - What worked best in monitoring the scale up process? - What would you do different in future scale up projects?   *(Examples: evaluation studies, formulating quality indicators)* |
| **Scale up success** | To what extent are you satisfied with the scale up of the intervention (so far)?  To what extent are/were scale up goals (if any) reached? |
| **Closing** | Anything to add/questions?  Thank you  Explain interview summary  Do you perhaps know anyone else we can interview about the scale up of a school-based mental health intervention?  Stop recording |
