# Supplementary material for: Analysis of real-world scale-up processes for school-based mental health interventions
Source: Adm Policy Ment Health. 2026 Mar 9;53(3):224–39. doi: 10.1007/s10488-026-01491-0 (PMC13221318; doi:10.1007/s10488-026-01491-0)
Supplement: Supplementary file 4 — Supplementary Material 4 [file 10488_2026_1491_MOESM4_ESM.pdf]

Theme	Definition	Subcode	Definition
Scale up barriers	Barriers for scaling up school-based mental health interventions.	Insufficient evidence	Barriers for scale up related to the evidence base of the intervention.
		Intervention target	Barriers for scale up related to the problem(s) targeted by the intervention, including stigma and taboo on mental
		Lack of politcal support	Barriers related to a lack of political support for scale.
		Lack of resources	Barriers related to a lack of resources for scale up, including financial resources.
		Personnel shortages	Barriers for scale up related to personnel shortages in the educational sector.
		Project involvement	Barriers for scale up related to the involvement of various stakeholders in the project, including changes in involvement.
		Scale up process	Barriers related to the process of scaling up
		Target population	Barriers for scale up related to the target population of the intervention.
		Time constraints	Barriers for scale up related to time
Scale up facilitators	Facilitators for scaling up school-based mental health interventions.	Evidence base	Facilitators for scale up related to the
		Financial support	Facilitators related to the availability of financial support for scaling up.
		Intersection of factors	Impact of the intersection of several facilitating factors on scale up.
		Low costs	Facilitators related to the costs of scaling up the intervention.
		Perceived need	Facilitators for scale up related to a perceived need for the intervention, for example to a perceived need among
		Problem urgency	Facilitators for scale up related to the perceived urgency of the problem the intervention targets within society.
		Support from institutions	Facilitators related to support for scale up from institutions, for example political support, managerial support, support from
Dissemination strategies	Strategies for spreading the intervention to new user settings.	Actively approaching	Actively approaching new user settings to
		Intervention registries	Inclusion of the intervention in intervention registries.
		Media	Spreading word about the intervention through media channels, including social
		Networks	Spreading word about the intervention through networks, for example educational networks or interest groups.

		Presenting	Presenting the intervention to the public (including but not limited to potential users, policy makers, target populations), for example workshops or seminars.
		Scientific dissemination	Spreading word about the intervention through scientific channels, for example scientific publications or conference
Strategies for organizing scale up	Strategies for organizing the scale up process.	Adaptations	Adapting the intervention to improve
		Collaboration	Collaborating with all kinds of partners to facilitate scale up.
		Communication streams	Communication streams between user settings and coordinators.
		Core team	A core team that coordinates the scale up of the intervention.
		Implementation support	Supporting new user settings in the implementation of the intervention.
		Intervention ownership	Strategies related to increasing intervention ownership on a setting level.
		Local teams	Formation of local intervention teams that support and coordinate implementation on
		Provision of materials	Providing necessary intervention materials to new user settings.
		Provision of training	Providing necessary trainings to new user
Financing strategies	Strategies for establishing financial resources for scale up.	Cost sharing	Sharing costs with any kinds of other
		Free materials	Offering necessary intervention materials to new user settings free of costs.
		Reducing implementation costs	Strategies to reduce implementation costs for potential user settings.
		Research funds	Establishing funds for follow-up research.
		Training funds	Establishing funds to be able to train deliverers in new user settings.
Strategies for monitoring and evaluation	Strategies for monitoring and evaluating the scale up process.	Effect evaluation	Evaluation studies into the effectiveness of
		Evaluation tools	Developing evaluation tools for new user
		Monitoring fidelity	Strategies for monitoring fidelity to the intervention in new user settings.
		Process evaluation	Evaluation studies into the scale up and/or implementation process of the
Other strategies	Strategies for scale up that do not fit with the previous categories.	Intervention development	Strategies for increasing scalability of the
		System changes	Strategies for increasing scalability of the intervention aimed at influencing systems, including but not limited to policies, legislation or guidelines.
