# Supplementary material for: Analysis of real-world scale-up processes for school-based mental health interventions
Source: Adm Policy Ment Health. 2026 Mar 9;53(3):224–39. doi: 10.1007/s10488-026-01491-0 (PMC13221318; doi:10.1007/s10488-026-01491-0)
Supplement: Supplementary file 5 — Supplementary Material 5 [file 10488_2026_1491_MOESM5_ESM.docx]

Table 1: Study participant characteristics

|  |  | Included publications  (n = 181) | Survey participants  (n = 45) | Interview participants  (n = 12) |
| --- | --- | --- | --- | --- |
| Country in which participant worked | Africa | 1 (1%) | 0 (0%) | 0 (0%) |
|  | Asia | 28 (15%) | 7 (16%) | 2 (17%) |
|  | Central America | 0 (0%) | 0 (0%) | 0 (0%) |
|  | Europe | 42 (23%) | 10 (22%) | 4 (33%) |
|  | North America | 71 (39%) | 15 (33%) | 4 (33%) |
|  | Oceania | 32 (18%) | 11 (24%) | 1 (8%) |
|  | South America | 7 (4%) | 2 (4%) | 1 (8%) |
| Age | 18-29 |  | 5 (11%) | 0 (0%) |
|  | 30-39 |  | 6 (13%) | 3 (25%) |
|  | 40-49 |  | 13 (29%) | 3 (25%) |
|  | 50-59 |  | 9 (20%) | 1 (8%) |
|  | 60+ |  | 12 (27%) | 5 (42%) |
| Work sector during intervention trial | University/academic |  | 37 (82%) | 7 (58%) |
|  | Mental health care |  | 6 (13%) | 3 (25%) |
|  | Other health care sector |  | 1 (2%) | 0 (0%) |
|  | NGO or non-profit |  | 6 (13%) | 2 (17%) |
|  | National mental health organization |  | 1 (2%) | 0 (0%) |
|  | Education |  | 1 (2%) | 1 (8%) |
| Current work sector | University/academic |  | 31 (69%) | 6 (50%) |
|  | Mental health care |  | 7 (16%) | 3 (25%) |
|  | Other health care sector |  | 1 (2%) | 1 (8%) |
|  | NGO or non-profit |  | 7 (16%) | 1 (8%) |
|  | Community mental health organization |  | 1 (2%) | 0 (0%) |
|  | Research consultancy |  | 2 (4%) | 0 (0%) |
|  | Other |  | 6 (13%) | 0 (0%) |
| Work experience in current sector | 1-5 years |  | 3 (7%) | 0 (0%) |
|  | 6-10 years |  | 14 (31%) | 6 (50%) |
|  | 11-15 years |  | 4 (9%) | 1 (8%) |
|  | 16-20 years |  | 6 (13%) | 1 (8%) |
|  | 21-25 years |  | 8 (18%) | 1 (8%) |
|  | >25 years |  | 10 (22%) | 3 (25%) |
| Current position | Academic |  | 32 (71%) | 5 (42%) |
|  | Mental health care professional |  | 6 (13%) | 3 (25%) |
|  | Senior manager |  | 2 (4%) | 0 (0%) |
|  | Other |  | 8 (18%) | 5 (42%) |
|  | Retired |  | 3 (7%) | 1 (8%) |
| Project role during intervention trial | Principal investigator |  | 26 (58%) | 7 (58%) |
|  | Project manager |  | 10 (22%) | 0 (0%) |
|  | Co-investigator |  | 2 (4%) | 2 (17%) |
|  | Phd student |  | 6 (13%) | 1 (8%) |
|  | Phd supervisor |  | 1 (2%) | 1 (8%) |
|  | Clinician |  | 1 (2%) | 0 (0%) |
|  | Other |  | 2 (4%) | 1 (8%) |
