# Supplementary material for: Analysis of real-world scale-up processes for school-based mental health interventions
Source: Adm Policy Ment Health. 2026 Mar 9;53(3):224–39. doi: 10.1007/s10488-026-01491-0 (PMC13221318; doi:10.1007/s10488-026-01491-0)
Supplement: Supplementary file 6 — Supplementary Material 6 [file 10488_2026_1491_MOESM6_ESM.docx]

**Additional supporting interview quotes for study “Learning from practice: analysis of real-world scale-up processes for school-based mental health interventions”**

Additional to the most relevant interviews quotes supporting our results given in the publication itself, we provide other supporting quotes in this document. The quotes are ordered per part of the results that they support, using the headings as given in the publication itself.

## Barriers and facilitators for scale-up

### Intervention characteristics

*“The results were not that convincing that we thought: ‘we have to continue’.”* – discontinued intervention 1, Europe

*“There are other evidence based prevention programs for depression […], but they are much longer […]. So when [potential implementers] go out and look, they see ‘hey, this program’.”* – success example 2, North America

*“[Our country] was going into a very difficult political situation back then […], the vice president started discontinuing a lot of research.”* – discontinued intervention 1, Europe

*“In the US we have a lot of funding now, even in more conservative states […], for public health education. There is a lot of need and people are coming for our type of solution.”* – success example 1, North America

### Resource availability

*“There’s some states where we have state level funding […], some states have even*

*prioritized our approach.”* – success example 1, North America

*“There is so many things to do, so many patients [in mental healthcare]. We didn’t have the power to scale-up on our own.”* – discontinued intervention 3, Europe

*“[My team] is really an organization of committed people.”* – success example 2, North America

### School context

*“We don’t have any room specifically for health education in our school curriculums.”* – in-progress example 2, Asia

*“[The schools] had no idea what I was talking about. And I think that is still an issue for any kind of mental health prevention in the school system.”* – success example 3, North America

## Scale-up strategies

### Domain 1: Strategies for dissemination and advocacy

*“There are three or four [intervention registries] we have been listed on. That has been a good way for people to find out about us, probably the best way*.” – success example 2, North America

*“[An intervention registry] included our intervention as one of their approved programs […], which was a very good mechanism for scale up.”* – success example 3, North America

### Domain 2: Strategies for organizational process

*“We are trying to make the program briefer or see if we could find other ways [for delivery], like who could provide it.”* – success example 2, North America

*“I have a three day workshop now, we’re going through a process of changing that. We are going to go hopefully online for scaling up.”* – success example 3, North America

*“We have about 10 people in the world who have been certified as trainers to train other people.”* – success example 2, North America

*“It’s really an organization of committed people who have gone through the more supervised training […], we have about 10 people who are part of the core group.”* – success example 3, North America

*“I have these nodes across different states and places where connections and little communities of [intervention] trainers developed.”* – success example 3, North America

### Domain 4: Strategies for evaluation and monitoring

*‘We ask them to video record, then my colleagues and I rate for adherence to the manual and how they do at delivering.”* – success example 2, North America

*“We’re doing mock-sessions […] so that the teachers will teach lessons to each other and videotape them, and then I look at the lessons and give them feedback.”* – success example 3, North America
